# The effect of decellularization protocols on characterizations of thermoresponsive and light-curable corneal extracellular matrix hydrogels

**DOI:** 10.1038/s41598-023-35202-8

**Published:** 2023-05-19

**Authors:** Ghasem Yazdanpanah, Elmira Jalilian, Xiang Shen, Khandaker N. Anwar, Yizhou Jiang, Sayena Jabbehdari, Mark I. Rosenblatt, Yayue Pan, Ali R. Djalilian

**Affiliations:** 1 Department of Ophthalmology and Visual Sciences, Illinois Eye and Ear Infirmary, University of Illinois at Chicago, 1855 W. Taylor Street, MC 648, Chicago, IL 60612 USA; 2 Department of Bioengineering, University of Illinois at Chicago, Chicago, IL USA; 3 Department of Mechanical and Industrial Engineering, University of Illinois at Chicago, Chicago, IL USA; 4 Jones Eye Institute, University of Arkansas for Medical Sciences, Little Rock, AR USA

**Keywords:** Gels and hydrogels, Corneal diseases

## Abstract

To compare the effects of two decellularization protocols on the characteristics of fabricated COrnea Matrix (COMatrix) hydrogels. Porcine corneas were decellularized with Detergent (De) or Freeze–Thaw (FT)-based protocols. DNA remnant, tissue composition and α-Gal epitope content were measured. The effect of α-galactosidase on α-Gal epitope residue was assessed. Thermoresponsive and light-curable (LC) hydrogels were fabricated from decellularized corneas and characterized with turbidimetric, light-transmission and rheological experiments. The cytocompatibility and cell-mediated contraction of the fabricated COMatrices were assessed. Both protocols reduced the DNA content to < 0.1 µg/mg (native, > 0.5 µg/mg), and preserved the collagens and glycosaminoglycans. The α-Gal epitope remnant decreased by > 50% following both decellularization methods. We observed more than 90% attenuation in α-Gal epitope after treatment with α-galactosidase. The thermogelation half-time of thermoresponsive COMatrices derived from De-Based protocol (De-COMatrix) was 18 min, similar to that of FT-COMatrix (21 min). The rheological characterizations revealed significantly higher shear moduli of thermoresponsive FT-COMatrix (300.8 ± 22.5 Pa) versus De-COMatrix 178.7 ± 31.3 Pa, *p* < 0.01); while, this significant difference in shear moduli was preserved after fabrication of FT-LC-COMatrix and De-LC-COMatrix (18.3 ± 1.7 vs 2.8 ± 2.6 kPa, respectively, *p* < 0.0001). All thermoresponsive and light-curable hydrogels have similar light-transmission to human corneas. Lastly, the obtained products from both decellularization methods showed excellent in vitro cytocompatibility. We found that FT-LC-COMatrix was the only fabricated hydrogel with no significant cell-mediated contraction while seeded with corneal mesenchymal stem cells (*p* < 0.0001). The significant effect of decellularization protocols on biomechanical properties of hydrogels derived from porcine corneal ECM should be considered for further applications.

## Introduction

Corneal transplantation is currently the main viable treatment option for restoring vision in severe corneal diseases or injuries. However, donor organs are available to less than 5% of new cases worldwide^1^. One of the approaches to overcome the issue of donor corneal tissue is developing tissue engineered corneas or in-situ regeneration protocols^2^. An emerging approach to fabricate the necessary biomaterials for corneal regeneration is to use decellularized extracellular matrix (ECM) from xenogeneic corneal tissues^3,4^. The advantages are the wider availability of xenogeneic tissue, and the inclusion of bioactive components that closely resembles the native tissue, particularly growth factors, cell adhesion proteins and glycosaminoglycans (GAGs)^5,6^. For tissue-engineering strategies aiming to replace the damaged organ, the intimate dynamic relationship between cells and the ECM is crucial for ensuring optimum tissue function^7^. The ECM macromolecules not only provide structural support, like a bio-scaffold, but also are biologically active and modulate important functional roles such as cell adhesion, migration, proliferation, differentiation, and survival^6,8–10^.

Decellularization of corneal matrix requires the removal of cellular and immunogenic components from the tissue while preserving the ECM with its native micro- and macroscopic anatomy^11^. Various protocols for decellularizing human and animal corneal tissues have been reported including detergents such as sodium dodecyl sulfate^12,13^, Triton x-100^6,14,15^, hypertonic saline^16^, high hydrostatic pressure^17,18^, freeze–thaw^19–21^, nitrogen gas^16,22^, phospholipase A2^23^, and glycerol^24^. Despite the availability of multiple decellularization techniques, there is still no standardized protocol for optimal decellularization of corneal tissues^25^.

Due to the shortage of human organs, and with recent advances in xenotransplantation, porcine organs owing to their similarity to humans’ physiology and organ size, have been considered as a suitable candidate for transplantation into human recipients^26^. However, there are some concerns associated with the immune response of pig-to-human tissue and organ transplantation. One of the major concerns is the α-Gal epitope that is ubiquitously present in non-primate mammals, marsupials and New World Monkeys, but it is absent in humans^27^. Binding of natural anti-pig antibodies in humans to α-Gal antigens expressed on the transplanted pig organ and tissues, activate the complement cascade, which results in destruction of the graft within minutes or hours, known as hyperacute rejection^28^. These have long been known to be major barriers for the survival of porcine derived natural products in humans. Therefore, removal of α-Gal epitope is essential to avoid immune system reactions and graft rejection^29^.

An evolving approach to utilize the produced decellularized corneal ECMs in corneal tissue engineering and regeneration is to turn the decellularized ECM into in-situ cross linking hydrogel^6^. We have generated an *in-situ* transparent, thermoresponsive hydrogel from COrneal Matrix (COMatrix) by decellularization and solubilization of decellularized porcine corneal ECM^6^. We have performed the rheological and compositional characterization on the fabricated thermoresponsive COMatrix^6^; and, also showed the potential of COMatrix as an ocular surface bandage to promote healing of corneal epithelial wounds^10^. Moreover, we recently fabricated a light-curable variant of decellularized porcine corneal extracellular matrix (LC-COMatrix)^30^. Characterization of LC-COMatrix showed excellent biomechanical and bio-adhesive properties of this hydrogel which could be crosslinked in-situ for repair of corneal defects and perforations. LC-COMatrix has a promising potential for repair of corneal perforations and corneal stromal defects in large animal models^30^. However, there are not enough studies evaluating the effects of various decellularization methods on the characterization of the fabricated hydrogel from decellularized corneas, especially the novel light-curable variant of these biomaterials.

**Figure 1:**
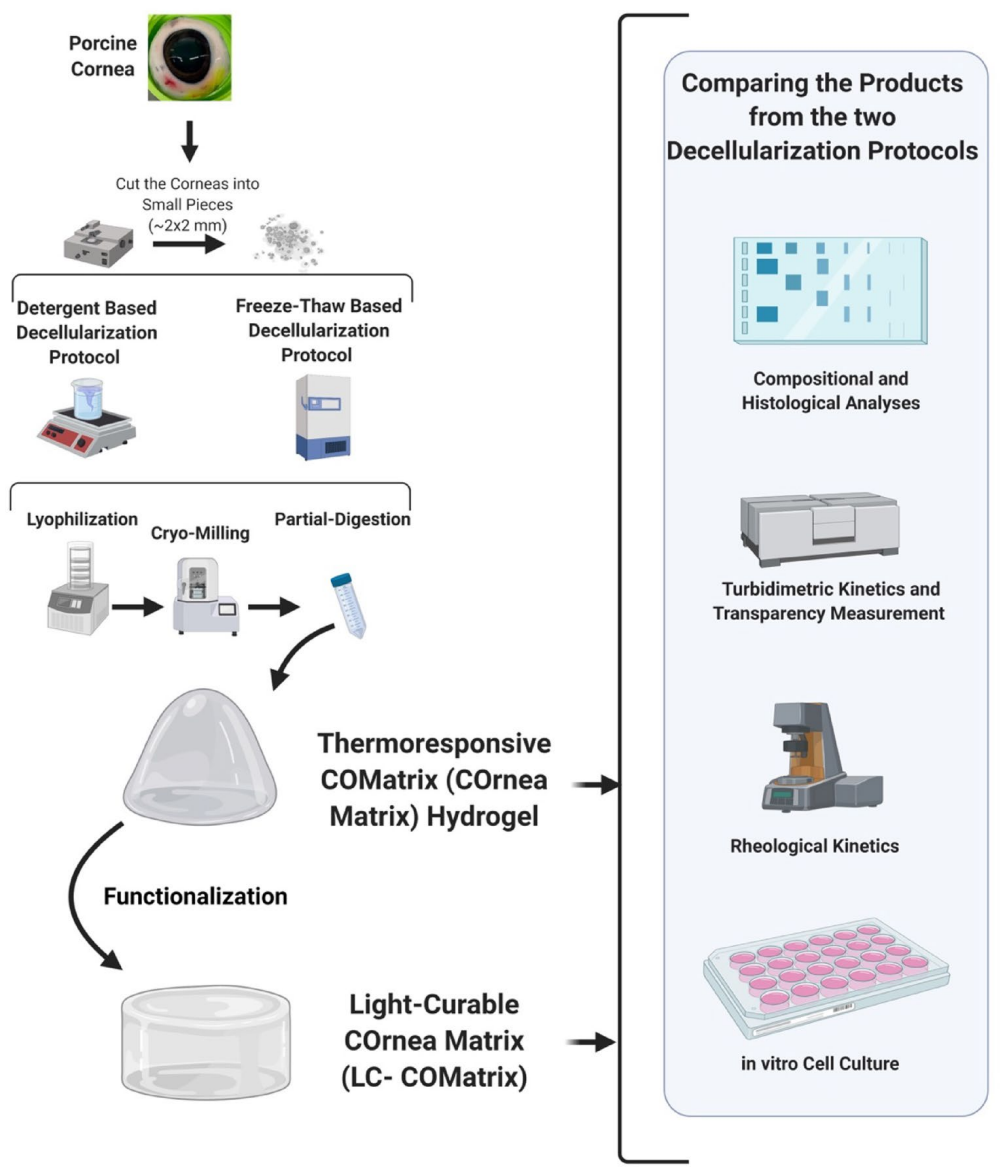
Illustration of the experiments in this study. Porcine corneas were first cut into small pieces and then decellularized with either Detergent-based (De) or Freeze–Thaw (FT) protocol. The decellularized tissues were freeze-dried, cryo milled and partially-digested to a hydrogel. The thermoresponsive hydrogels from both decellularization methods (detergent-based: De-COMatrix and freeze–thaw: FT-COMatrix) were functionalized to be able to be cured with visible light for gelation. The light-curable COMatrices were designated as De-LC-COMatrix and FT-LC-COMatrix. Characterizations included compositional and histological analyses, turbidimetric kinetics and transparency measurements, rheological kinetics and cytocompatibility assays.

In this study we examined the impact of two different decellularization protocols on the properties of produced COMatrix hydrogels from porcine corneas (Fig. 1). The first protocol is a non-ionic detergent (De)-based decellularization technique and the second is a freeze–thaw (FT) cycling method to decellularize porcine corneas. The efficiency of decellularization protocols in removing the cell and DNA remnants and preserving the collagen and sulfated glycosaminoglycan contents was evaluated. Moreover, the extent of remained α-Gal epitope was assessed with immunofluorescence staining and western blot, in addition to determining the effectiveness of α1-3,6 galactosidase enzyme treatment for more efficient removal of α-Gal epitope. Then, two types of COMatrix hydrogels were fabricated from the resultant decellularized porcine corneal ECMs; thermoresponsive and light-curable (LC). The impacts of the decellularization protocols on the biochemical composition, transparency, gelation kinetics, mechanical properties, cytocompatibility and cell-mediated contraction of the manufactured COMatrices were evaluated.

## Materials and methods

All the performed experiments in this study are in accordance with the guidelines of the Office of the Vice Chancellor for Research at The University of Illinois at Chicago.

### Decellularization of porcine corneas

Fabrication of COrneal Matrix (COMatrix) hydrogel requires decellularization and solubilization of corneal tissues. Under sterile conditions, porcine corneas (PC) were dissected from fresh, intact porcine eyeballs that were obtained from a certified abattoir (Park Packing Co. Inc., Chicago, IL). The extracted PCs were then washed with phosphate buffered saline (PBS, 1×) containing 1% gentamicin, 1% penicillin and 1% streptomycin. Decellularization of porcine corneas were performed using two different methods, Detergent-based (De) or Freeze–Thaw (FT). The De-based decellularization of PCs was performed as described before^6^. PCs were cut into 2 × 2 mm^2^ pieces; tissue pieces were initially stirred in 20 mM ammonium hydroxide solution (Sigma, USA) containing 0.5% Triton X-100 (Fisher Scientific, USA) in distilled water (pH 10) for 4 h at room temperature. Tissues were then transferred to 10 mM Tris–HCl (Sigma, USA) containing 0.5% EDTA (Fisher Scientific, USA) in distilled water (pH 7.5) and continuously stirred for 24 h at room temperature. PC tissues were then continuously stirred at 37 °C for 24 h in 10 mM Tris–HCl containing 1% (v/v) Triton X-100 (pH 7.5).

In the FT decellularization protocol, the small pieces (2 × 2 mm^2^, 20–25 corneas) of porcine corneas were put in a 50 ml tube containing 35 ml of 10 mM Tris–HCl plus protease inhibitor cocktail (cOmplete™, EDTA-free Protease Inhibitor Cocktail, Roche) and put in a − 80 °C freezer for a minimum of 5 h. Thereafter, the tube was placed at room temperature till full thawing. The freeze–thaw cycles were repeated for 9 times. Then, the tissue pieces were washed with pure water for 1 day.

### DNA remnant removal and bio-burden reduction

To remove DNA remnants from decellularized porcine cornea, the tissue fragments were agitated for 16 h at 37 °C, in 50 mM Tris–HCl containing 7.5 U/ml deoxyribonuclease (Sigma, USA) in molecular biology grade water (pH 7.5, Fisher Scientific, USA). The samples were then stirred in PBS (pH 7.5) for 48 h at room temperature; PBS was changed twice per day. Bioburden of decellularized PC was reduced by stirring in 0.1% peracetic acid (32 wt% in dilute acetic acid, Sigma, USA) in 4% ethanol in molecular biology grade water, at room temperature, for 20 h. Lastly, tissues were stirred in molecular biology grade water, three times, each for two hours. Samples were taken at this stage to assess efficacy of the decellularization process (see below). Tissues were snap-frozen in liquid nitrogen for 48 h and then lyophilized at − 55 °C and < 0.133 mBar. Lyophilized tissues were then stored at − 80 °C until needed and for no more than 6 months. Non-decellularized corneas were lyophilized as well to be used as control (native porcine corneas).

### α1-3,6 galactosidase treatment

To evaluate the effects of α1-3,6 Galactosidase treatment on the α-Gal epitope removal from porcine corneas, the tissues already decellularized with FT method were treated with α1-3,6 Galactosidase enzyme (2.5 U/ml, New England Biolabs) while incubating with DNase (see the DNAse treatment protocol above). The rest of the protocol was the same as above.

### Histological assessment

The decellularized porcine cornea tissues were evaluated by various tissue staining including hematoxylin and eosin (H&E), Alcian blue, and Picro Sirius Red staining. The decellularized tissue pieces were fixed in the Tissue-Tek® optimum cutting temperature (O.C.T.) solution and frozen on dry ice. After sectioning using Cryostat (Fisher Scientific, USA) and transferring to histological slides, the sections were fixed in 4% paraformaldehyde for 15 min and washed with deionized (DI) water. For H&E staining, the slides were incubated in hematoxylin (Fisher Scientific, USA) for 2 min followed by washing with DI water. After that, the slides were briefly treated with 70% Ethanol/1% HCl and washed with DI water. The slides were then stained with eosin (Fisher Scientific, USA) for 7 min and then washed briefly with 95% ethanol. To evaluate the collagen distribution, Picro-sirius red staining was performed. Slides were incubated with Sirius Red (Sigma-Aldrich) in a saturated aqueous solution of picric acid for 1 h. Then they were rinsed for one minute in 0.5% acetic acid. To visualize the sGAG content with Alcian blue staining, slides were stained with 1% Alcian Blue 8GX (Sigma-Aldrich) in 0.1M HCl for 5 min. Then, the slides were washed three times in deionized H2O, each for 30-s. After all staining, the slides sections were dehydrated with 100% ethanol for 10 min followed by incubating in xylene (Fisher Scientific, USA) for 10 min. After drying at room temperature, one drop of permount (Fisher Scientific, USA) was applied to the slide and a coverslip (22 × 40 mm) was placed on the tissue sections and sealed with nail polish. The stained tissue sections were visualized and imaged with a light microscope (Zeiss, Germany).

### Immunostaining

The tissue sections (see above for sectioning the tissues) were first fixed with 4% paraformaldehyde for 15 min at room temperature. They were then blocked at room temperature with 5% bovine serum albumin (Sigma, USA). Then sections were incubated with primary antibody, mouse α-Gal Epitope (Galα1-3Galβ1-4GlcNAc-R) monoclonal IgM antibody (M86, Enzo Life Sciences) with concentration of 1:5 and incubated at 4° C overnight. Sections were then washed 3× with PBST (PBS + 0.1% Tween-20) and then incubated with goat anti-mouse IgM Alexa Fluor 488-conjugated secondary antibody (Thermo Fisher) for 1 h at room temperature. Sections were then washed with PBST 3 times and mounted with ProLong™ Gold Antifade Mountant with DAPI (Thermo Fisher). Then, sections were imaged using a confocal microscope (LSM 710, Carl Zeiss, Germany). The Images were analyzed with ZEN Lite software (Zeiss, Germany).

### Thermoresponsive COMatrix hydrogel fabrication

To fabricate thermoresponsive COMatrix hydrogel from decellularized or native porcine corneas; first, the lyophilized tissue pieces were cryo-milled using a freezer-mill (Spex 6700, USA). The resultant fine powder was sieved using a mesh (size 40, Sigma, USA) and partially digested by stirring in 0.01M HCl (20 mg/ml) containing 1 mg/ml pepsin (> 400 U/mg, Sigma, USA) for 72 h at room temperature. Then, the digested PC-ECM was neutralized to pH 7 using one:ninth 0.1 M NaOH and one: tenth PBS (10×), while on ice. The resulting hydrogel was diluted to desired concentrations using PBS (1×). To induce thermogelation, the cool COMatrix hydrogel was incubated at 37 °C for 30–45 min. The thermoresponsive COMatrix hydrogels derived from detergent-based decellularization protocol called, De-COMatrix; and thermoresponsive hydrogels derived from freeze–thaw decellularization method called, FT-COMatrix.

### Light-curable COMatrix hydrogel fabrication

To fabricate light-curable (LC) COMatrix hydrogel from the porcine corneas decellularized with detergent-based or freeze–thaw methods, the already produced thermoresponsive COMatrix hydrogels were reacted with methacrylate anhydride (Sigma, USA) at 4 °C in the dark for 12 h, as previously reported^30^. Then, the samples were dialyzed against deionized water for 3 days at room temperature using 12–14 kDa MWCO dialysis tubes. The functionalized COMatrix was freeze-dried for 3 days and kept in − 80 °C for further experiments. To prepare the LC-COMatrix solution for experiments, the lyophilized samples were dissolved in a photo-initiating cocktail including Eosin Y, Ethanolamine, and N-Vinylcaprolactam as described before^30^. Afterward, the LC-COMatrices were cured with green light (520 nm wavelength) to induce photogelation. The light-curable COMatrix hydrogels derived from detergent-based decellularization protocol called, De-LC-COMatrix; and, light-curable hydrogels derived from freeze–thaw decellularization method called, FT-LC-COMatrix.

### Biochemical quantification of COMatrix hydrogel

Relevant assays to measure amounts of DNA, total collagen and sulfated glycosaminoglycans (sGAGs) in COMatrix hydrogel (pepsin digested decellularized ECMs) were performed^6^. Samples were digested in papain extraction solution (1:1 v/v, 50 ml of 0.2 sodium phosphate buffer (pH 6.4), 400 mg sodium acetate, 200 mg EDTA, 40 mg cysteine HCl, 250 µl of papain suspension (Sigma, p3125) containing 5 mg of enzyme overnight at 65° C. Genomic double-strand DNA were purified using a commercially available kit (catalog #G1N70, Sigma, USA) to quantify DNA content of samples, following the manufacturer protocol. DNA concentrations were then measured using NanoDrop Microvolume Spectrophotometer (Fisher Scientific, USA) at 260 nm.

Total collagen content was measured using the hydroxyproline assay^6^. In brief, 100 µl of papain digested COMatrix hydrogel was added to 100 µl of 4 N NaOH separately, then hydrolyzed by autoclaving for 15 min. After cooling the samples to room temperature, 100 µl of 4 N HCL was added. Then, 100 µl of the resultant solution was mixed with 100 µl of chloramine-T solution and incubated for 20 min at room temperature. After that, 200 µl of p-dimethylaminobenzaldehyde (p-DAB) solution was added to the mixture, the tubes were incubated at 60 °C for 30 min, then quenched in room temperature water for 5 min. A 100 µl sample of the prepared mixture was transferred to a 96 well plate in triplicate, and the mixture absorbance was measured at 540 nm. Hydroxyproline dissolved in sodium phosphate buffer with concentrations of 200, 100, 50, 25, 12.5, 6.25, 3.125, 1.156 and 0 mg/ml was used to draw the standard curve.

We measured sGAG content using 1,9-dimethyl methylene blue (DMMB) assay. DMMB working solution was the combination of 5 ml of formate solution (0.25 g sodium formate in 24 ml of 1 M guanidine hydrochloride (GuHCl) and 0.2975 ml of > 95% formic acid), 12.5 ml 200 proof anhydrous ethanol, 6.4 mg of DMMB (Sigma, USA), and 7.5 ml ultrapure water. 100 µl of papain digested ECM or standard was added to 1 ml of DMMB working solution, agitated at 150 rpm for 30 min in room temperature, and centrifuged to precipitate a sGAG-dye complex. The supernatant was aspirated, and 1 ml decomplexation solution was added to the pellet, before being agitated, again, at 150 rpm for 30 min at room temperature. The samples were transferred to a 96 well plate in triplicate, and absorbance was measured at 650 nm. Serial dilutions of chondroitin sulfate (Sigma, USA) from 200 µg/100 µl to 0 µg/100 µl was used as standards.

### SDS-PAGE and western blot

To evaluate the quality of fabricated COMatrix hydrogels derived from De- and FT-decellularization protocols, the samples were compared to pepsin-digested bovine tendon collagen using SDS-Page electrophoresis. 7.5 µl of each sample (6 mg/ml) was mixed with 3.75 µl LDS 4× Sample Buffer, 1.5 µl 10 × Reducing Agent (dithiothreitol), and 2.25 µl DI H2O (all are NuPAGE, Fisher Scientific, USA) to a total volume of 15 µl. The samples were vortexed and spun down and then were heated at 95 °C in a heating block for 15 min. Next, the samples were loaded into a 4–12% Bis–Tris gel (10 wells, 12 µl per well, NuPAGE, Fisher Scientific, USA) and subjected to electrophoresis using SDS running buffer (NuPAGE, Fisher Scientific, USA) and constant voltage (150 V) until the dye reached the bottom of the gel (~ 1.5 h). Then, deionized water (3 × 5 min) was used to wash the gel and stained with Bio-Safe Coomassie Brilliant Blue G-250 (50 ml per gel, BIO-RAD, USA) for 1 h with gentle agitation. To visualize distinct bands the stained gel was rinsed with deionized water for 30 min.

For western blot experiments, same concentrations of samples separated in SDS-Page as described above. Then, the protein bands were transferred to polyvinylidene difluoride membranes. The membranes were gently agitated in 5% BSA in tris-buffered saline (TBS) for 1 h at room temperature. The membranes were then gently shaked in 5% BSA in TBS containing primary antibody at 4 °C overnight. To detect the keratocan protein, the polyclonal goat anti-keratocan (Santa Cruz Biotechnology, USA) was used with dilution of 1:1000. To detect the α-Gal epitope, the mouse monoclonal (M86) anti-Galα1-3Galβ1-4GlcNAc-R (Enzo Life Sciences) was used with dilution of 1:5. After the overnight incubation with primary antibodies, the membranes were washed with TBS containing 0.03% Tween-20 for 3 times (each 10 min) and then incubated with the horseradish peroxidase–conjugated secondary antibodies (1:10,000 dilution, Thermos Fisher) for 1 h at room temperature followed by 4 rounds of washing with TBS/Tween-20 solution. At the end, the membranes were imaged with a commercial detection system (ECL Plus Western Blotting Detection System; Amersham, Buckinghamshire, UK) using ImageQuant LAS 4000 series (GE, USA). All data were analyzed in a semi-quantitative way using imageJ for western blot and data were normalized based on negative controls. Native cornea was not decellularized, but was fully processed, and was used as a positive control and the reference for analysis. The unprocessed photos of western blots are shown in Supplementary information.

### Ultra-structural characterization of thermoresponsive COMatrix hydrogel

The ultra-structure of thermogelated COMatrix hydrogels was observed using scanning electron microscopy (SEM). 5, mg/ml samples were fixed with cold glutaraldehyde overnight and then dehydrated with serial dilutions of ethanol/hexamethyldisilazane (2:1, 1:1, 1:2 and 0:1, respectively, each step 30 min and allowed to evaporate in the last step). After that, the samples were sputter coated and visualized using SEM (JEOL JSM-IT500HR FESEM, USA).

### Turbidimetric assay for gelation kinetics of thermoresponsive COMatrix hydrogels

Gelation kinetics was determined via turbidimetric spectrophotometric analysis. This technique is based on increased turbidity, and thus absorbance, experienced during hydrogel self-assembly. The sol–gel transition (thermogelation) of the thermoresponsive COMatrix hydrogels when heated to 37 °C was characterized by turbidimetric assay. In this assay, 160 µl of 25 mg/ml cool (4 °C) COMatrix hydrogel was loaded cold in a 96 well plate. The experiment was performed in triplicate and repeated three times. Then, the plate was placed in a pre-warmed (37 °C) plate reader and absorbance at 405 nm was measured every 2 min, for 30 min. Absorbance values were normalized with the following formula^31^:

*NA* = (*A* − *A*_0_)/(*A*_*max*_ − *A*_0_).where NA is the normalized absorbance, A is the absorbance at any given time, A0 is the initial absorbance and Amax is the maximal absorbance. The time needed to reach 50% of the maximum turbidity measurement (e.g. maximum absorbance value) was defined as t_1/2_. The lag phase (t_lag_) was calculated by obtaining the linear portion of the curve and extrapolating the time value at which the normalized absorbance is 0. Similarly, t _1/2_ was determined as the time at which the normalized absorbance is 0.5. The speed (S) of the gelation based on turbidimetric measurements was determined by calculating the maximum slope of the growth portion of the curve.

### Light transmission measurements

To measure transparency of fabricated thermoresponsive COMatrix hydrogels as the results of two different decellularization protocols, 160 µl of 4 °C De-COMatrix or FT-COMatrix hydrogels at concentrations of 25 mg/ml (each triplicate) was loaded into a 96 well plate (~ 500 µm thickness). The thermogelation was induced and completed by incubating at 37 °C for 30 min. Light absorbance of each well was then measured at 300 to 800 nm, in 50 nm increments, in a plate reader pre-warmed to 37 °C (BioTek™ Synergy™ H1 Hybrid, USA). The absorbance of the same amount of PBS was deducted from recorded values. Then, the light transmission was calculated by using the following formula:

$${\text{Light Transmission }}\left( \% \right) = {\text{antilog }}({2} - {\text{absorbance}})$$

The same process was performed to measure the light-transmission of light-curable COMatrices (De-LC-COMatrix and FT-LC-COMatrix) cured with green light for 4 min.

### Rheological kinetics and characterization of thermoresponsive and light-curable COMatrix hydrogels

We used a rotational rheometer (Kinexus Ultra+, Malvern) with a parallel 25 mm plates and temperature controller to record the thermogelation course of COMatrix hydrogels^6^. The initial temperature of the rheometer bed was set to 12 °C, assuming gelation is negligible at this temperature. COMatrix hydrogel in 25 mg/ml concentration was loaded. The parallel gap was set to 0.9 mm and to prevent sample dryness, mineral oil was applied and trimmed around the plate for the duration of the experiment. The rheological indexes were measured with 0.159 Hz (1 rad/s) frequency and 0.5% strain. After recording for three minutes, the temperature was rapidly increased to 37 °C (in 25 s) to induce gelation. We recorded gelation data for 50 min to characterize kinetics and measure the rheological shear moduli (elastic, G′ and viscous, G″ shear moduli). At least, three different samples were characterized for each decellularization group.

Regarding light-curable hydrogels (De-LC-COMatrix and FT-LC-COMatrix), a glass plate was used to settle the hydrogel on top and a light source was used from bottom^30^. The parallel gap was set to 0.4 mm. The shear characteristics were started to record at 0.159 Hz (1 rad/s) frequency and 0.5% strain for 1 min, and then the green light (100 µW/cm^2^) switched on (at minute 1), and the photogelation started while recording the shear moduli. The light was turned off at minute 5 (total 4 min light curing) and recording was continued for another 6 min.

### Human corneal mesenchymal stem cells (MSCs) harvesting

Human corneal MSCs were isolated and expanded as described before by our team^32–35^. Human cornea tissue was generously provided by the Eversight eye bank (Ann Arbor, MI) from healthy cadaver eyes. Utilizing of cadaveric human corneas provided by the eye bank for research purposes has been in accordance of guidelines of the Officeof the Vice Chancellor for Research at the of University of Illinois at Chicago. In brief, human corneas were washed with 1× PBS for 5 times. PBS contained 2× antibiotic–antimycotic and 2× penicillin–streptomycin. After removing the sclera/conjunctiva, the central cornea was cut with an 8-mm trephine. The remaining rings including corneoscleral rim were cut into four fragments, and each fragment was plated in one well of a six well plate with 200 μl of MEM-α media supplemented with 10% FBS, 1% penicillin/streptomycin, 1% l-glutamine, and 1% NEAA on top of each explant, with epithelial surface upward to prevent detachment. The plates were then incubated in a humidified incubator with 5% CO_2_ at 37 °C until the cells started to outgrow. The outgrown cells were detached using 0.05% TrypLE (Thermo Fisher Scientific, Waltham, MA) and re-plated in a T-175 flask (P1) with the same medium. The media were changed every 2–3 days until confluent^36^. The human corneal MSCs at passages 3 and 4 were used for Cytocompatibility studies.

### In vitro cytocompatibility and cell-mediated contraction of hydrogels

The human corneal MSCs obtained from human cadaveric corneas (see above) were used to evaluate the biocompatibility of the fabricated thermoresponsive and light-curable COMatrix hydrogels derived from De- and FT-decellularization protocols. Gelation was induced either thermally by adding 200 µl of cold thermoresponsive COMatrix hydrogel into one well of a 48 well plate (~ 300 µm thickness) and incubation for 45 min at 37 °C; or by green light-curing of light-curable COMatrix hydrogels (200 µl, 300 µm) in 48 well plate for 4 min. Then, MSCs (4 × 10^3^ cells/well) were seeded on top of the hydrogels, and 350 µl medium added in each well. The plates were incubated in a humidified incubator with 5% CO_2_ at 37 °C. To measure the cell-mediated contraction of the fabricated thermoresponsive and light-curable COMatrices, the sizes of hydrogels seeded with MSCs were followed by scanning the plates using a document scanner (Epson) over different time points (Day 0, 9, 18 and 28).

The viability of MSCs was evaluated on day 28 of culture. The cells were stained with Calcein-AM (live cells), propidium iodide (PI, dead cells) and Hoechst 33342 (total cells, all from Sigma, USA) and incubated for 1 h at 37 °C in humidified atmosphere with 5% CO_2_. Then, the seeded cells on COMatrices were imaged using ZEISS Cell Observer SD Spinning Disk Confocal Microscope (Zeiss, Germany), and the images were analyzed using ZEN Lite software (Zeiss, Germany).

### Statistical analyses

Data are shown as mean ± standard deviation (SD). Statistical analyses were done by GraphPad Prism software version 8.3.0 (538) for Windows, (GraphPad Software, San Diego, California, USA, www.graphpad.com) using Student’s t-test for comparing the means between two groups and one-way analysis of variance (ANOVA) and Tukey posttest for more than two groups. *p* Values less than 0.05 were considered as statistically significant difference between groups.

### Translational relevance

This study shines the light on the significance of decellularization protocols on crucial characterizations of biomaterials fabricated from decellularized xenogeneic corneas even after secondary functionalization, which is very important for future clinical translation.

## Results

### Detergent and freeze–thaw -based decellularization methods have similar effects on histology and composition of porcine corneas/COMatrices

**Figure 2:**
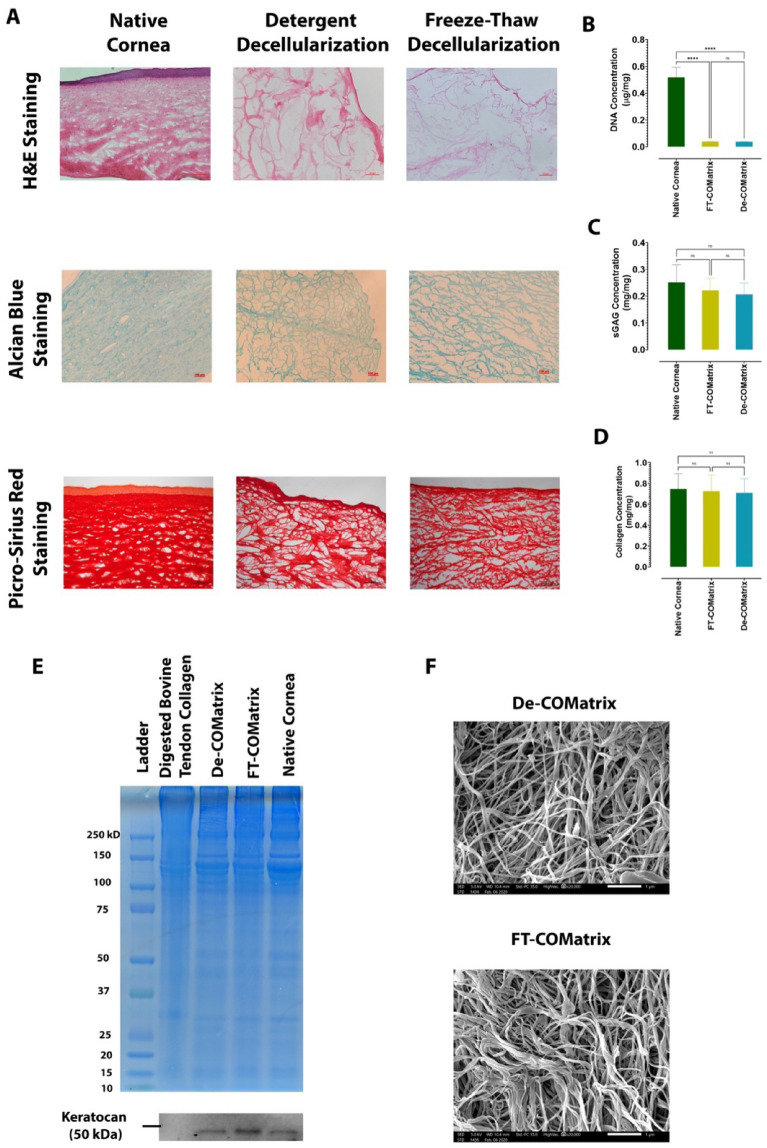
Evaluating the results of the two decellularization protocols: (**A**) histological examination of decellularized porcine corneas compared to native cornea, stained with haematoxylin and eosin (first row), Alcian blue (second row), picro-sirius red (third row); (**B**) Quantification of DNA remnant, (**C**) measurement of sGAG, and (**D**) collagen contents; (**E**) Protein composition of fabricated De- and FT-COMatrices using SDS-PAGE; (**F**) SEM microscopy of both FT-COMatrix and De-COMatrix. ****p < 0.0001.

We performed several experiments that compared the histology and composition of corneas decellularized with detergent-based or freeze–thaw methods. Hematoxylin and eosin (H&E) staining showed successful removal of corneal epithelial and stromal cells in both techniques (Fig. 2A, first row). We also measured the DNA remnant in decellularized samples which confirmed significant reduction of DNA in both utilized protocols (*p* < 0.0001, Fig. 2B). Alcian blue staining showed the presence of sGAG in both decellularized tissues which were similar to native cornea (Fig. 2A, second row). Both decellularization methods did not considerably change the total content of sulfated glycosaminoglycan (sGAG) in either FT-COMatrix and De-COMatrix as compared to native cornea. sGAG concentration was 0.25 ± 0.06 mg, 0.22 ± 0.04 mg and 0.20 ± 0.04 mg per each mg of tissue in native cornea, FT-COMatrix and De-COMatrix, respectively (*p* = 0.48, Fig. 2C). Lastly, the concentrations of total collagen in native cornea, FT-COMatrix and De-COMatrix after decellularization were 0.74 ± 0.15 mg, 0.72 ± 0.15 mg and 0.70 ± 0.13 mg per each mg of decellularized tissue, respectively (*p* = 0.95, Fig. 2D). Likewise, picro-sirius red staining also showed that the collagen fibers were not influenced by decellularization protocols (Fig. 2A, third row). In all experiments no significant difference was observed between the two decellularization techniques.

We performed further analysis of the composition of decellularized samples by using SDS-PAGE electrophoresis. As presented in Fig. 2E, the protein bands are similar in both hydrogel samples from different decellularization methods with native cornea. Also, it is clear that hydrogels from decellularized and digested porcine corneas are richer in the variety of proteins compared to digested bovine tendon collagen. Moreover, Keratocan, a protein rich in cornea with significant corneal healing effect, was conserved in both decellularization methods (Fig. 2E, western blot results). Furthermore, we used SEM to visualize the ultrastructure of the hydrogels, which confirmed that the porous and fibrillary structure of hydrogels were similar following both decellularization methods (Fig. 2F).

### α-Gal epitope was significantly decreased after porcine cornea decellularization

**Figure 3:**
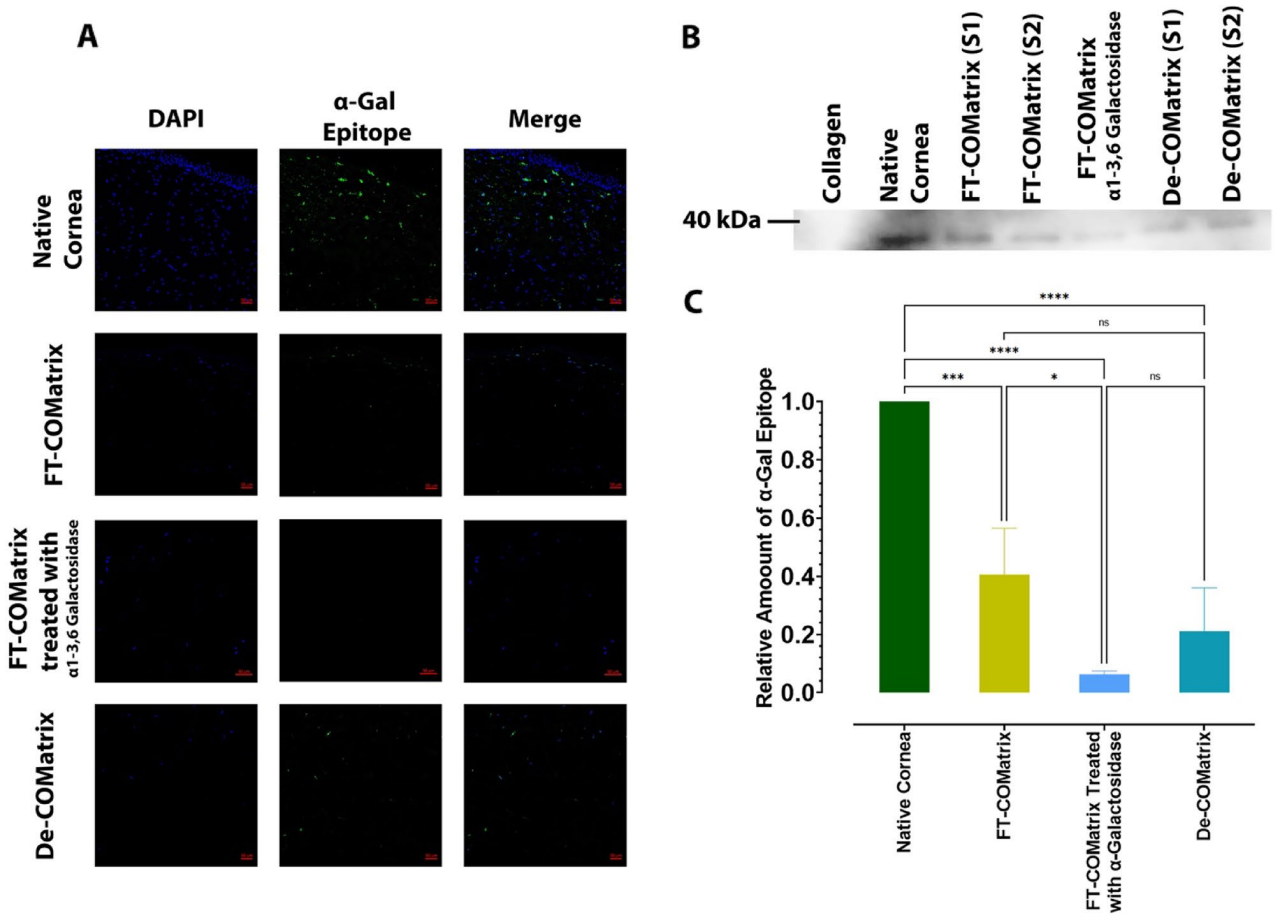
Measuring the remnant of α-Gal epitope after decellularization with detergent-based and freeze–thaw methods and also after treatment with α1-3,6 Galactosidase at the end of decellularization. (**A**) Immunostaining using anti-α-Gal antibody (M86) on native porcine cornea, detergent-based decellularized cornea, freeze–thaw decellularized porcine cornea and porcine corneas treated with α1-3,6 Galactosidase enzyme at the end of decellularization. The presented samples in this figure were not treated with DNAse for better localization of the cells. (**B**) Western blot result of α-Gal epitope content in hydrogels derived from different decellularization and treatment protocols. (**C**) Semi-quantitative measurement of α-Gal epitope remnants. The amount of α-Gal epitope in decellularized samples were normalized to the collagen at negative control and native cornea content of α-Gal epitope was considered as reference. ns, not significant; *, *p* < 0.05; ***, *p* < 0.001; ****, *p* < 0.0001.

We measured the α-Gal content before and after decellularization using immunofluorescence staining and western blot. Immunofluorescence staining showed that both freeze–thaw and detergent-based decellularization methods had removed the α-Gal epitope to some extent compared to native cornea. However, no α-Gal epitope was detected in the porcine corneas treated with α1-3,6 Galactosidase at the end of decellularization process (Fig. 3A). Moreover, measuring the relative amount of α-Gal epitope by western blot showed that detergent-based and freeze–thaw decellularization methods had significantly removed α-Gal epitope compared to native cornea (*p* < 0.001). Treatment with α1-3,6 Galactosidase further decreased the concentration of α-Gal to barely detectable levels (*p* < 0.0001, Fig. 3B and C).

### FT-COMatrix and De-COMatrix hydrogels have similar gelation kinetics and light transmittance

**Figure 4:**
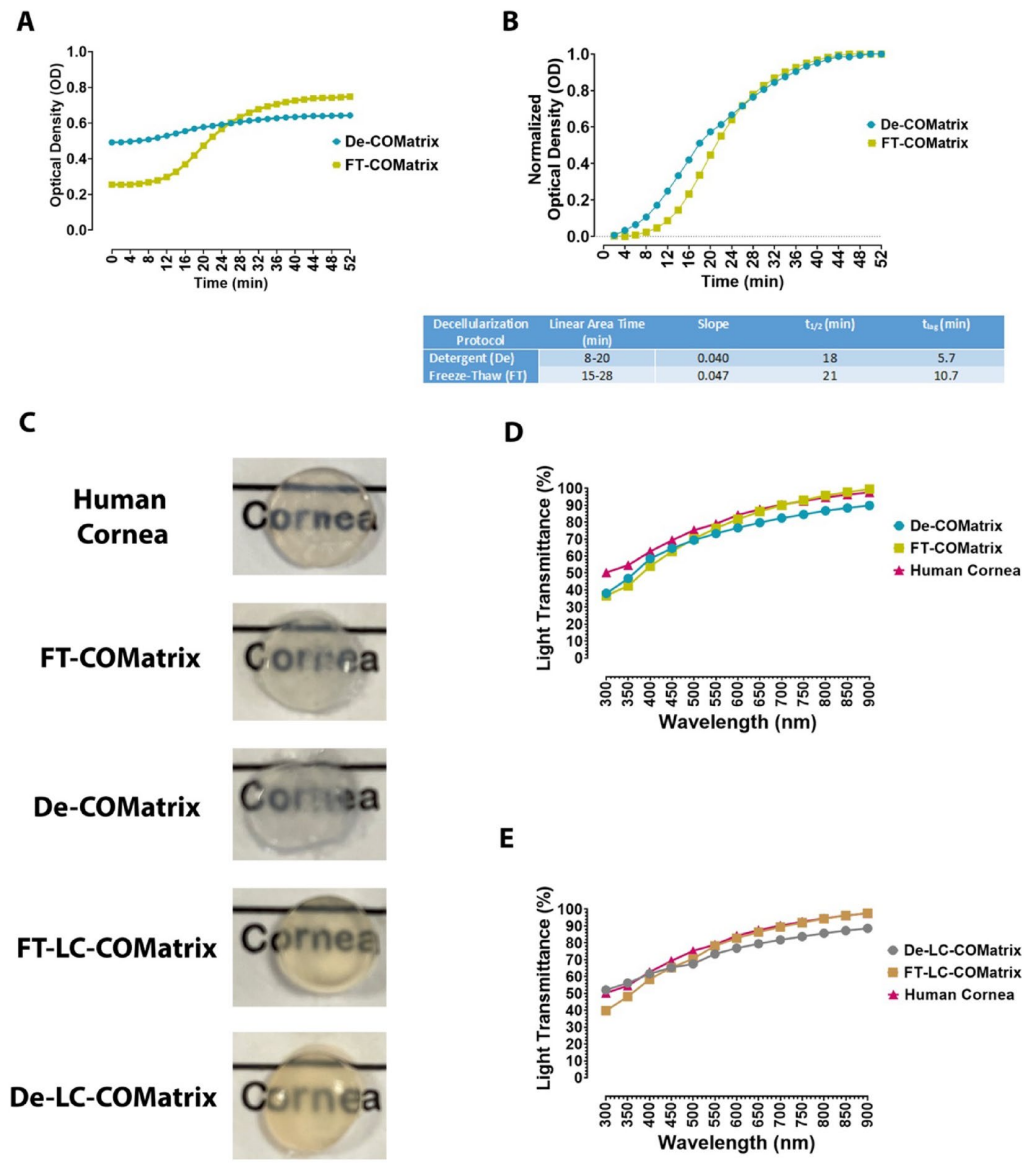
Gelation kinetics and transparency of fabricated COMatrices compared to human cornea: (**A**) Raw values for thermogelation turbidimetric kinetics of De- and FT-COMatrices; (**B**) Normalized turbidimetric kinetics and the thermogelation kinetics’ values (See methods for more detail); (**C**) Macroscopic appearance of 7 mm diameter buttons from human cornea, FT-COMatrix, De-COMatrix, FT-LC-COMatrix and De-LC-COMatrix placed over printed text; (**D**) Light transmittance (300 to 800 nm) of thermogelated De-COMatrix and FT-COMatrix compared to human cornea; (**E**) Light transmittance (300 to 800 nm) of photogelated De-LC-COMatrix and FT-COMatrix compared to human cornea (all samples are 500 µm in (**C**),(**D**), and (**E**)).

We analyzed the gelation kinetics of both thermoresponsive hydrogels by turbidimetric analysis and the results illustrated in Fig. 4A. FT-COMatrix presented more sigmoidal profile compared to De-COMatrix; however, the gelation kinetics of both hydrogels looked more similar after normalization (Fig. 4B). Although, the thermogelation of De-COMatrix started (t_lag_, 5.7 min) earlier than FT-COMatrix (t_lag_, 10.7 min), there was no statistically significant difference in kinetics of gel formation between the two decellularization methods (both hydrogels have similar linear area time, slope, and gelation half-time, t_1/2_).

As presented in Fig. 4C, all thermoresponsive and light-curable hydrogels allowed light to pass through them following thermogelation similar to human cornea (500 µm thickness for all samples). Both thermoresponsive and light-curable hydrogels derived from detergent-based decellularization technique were slightly less transparent to the naked eye, although quantitative light transmittance measurements using spectrophotometer did not show a significant difference between various hydrogels and human cornea (Fig. 4D and E).

### COMatrix derived from freeze–thaw decellularization has higher rheological shear moduli

**Figure 5:**
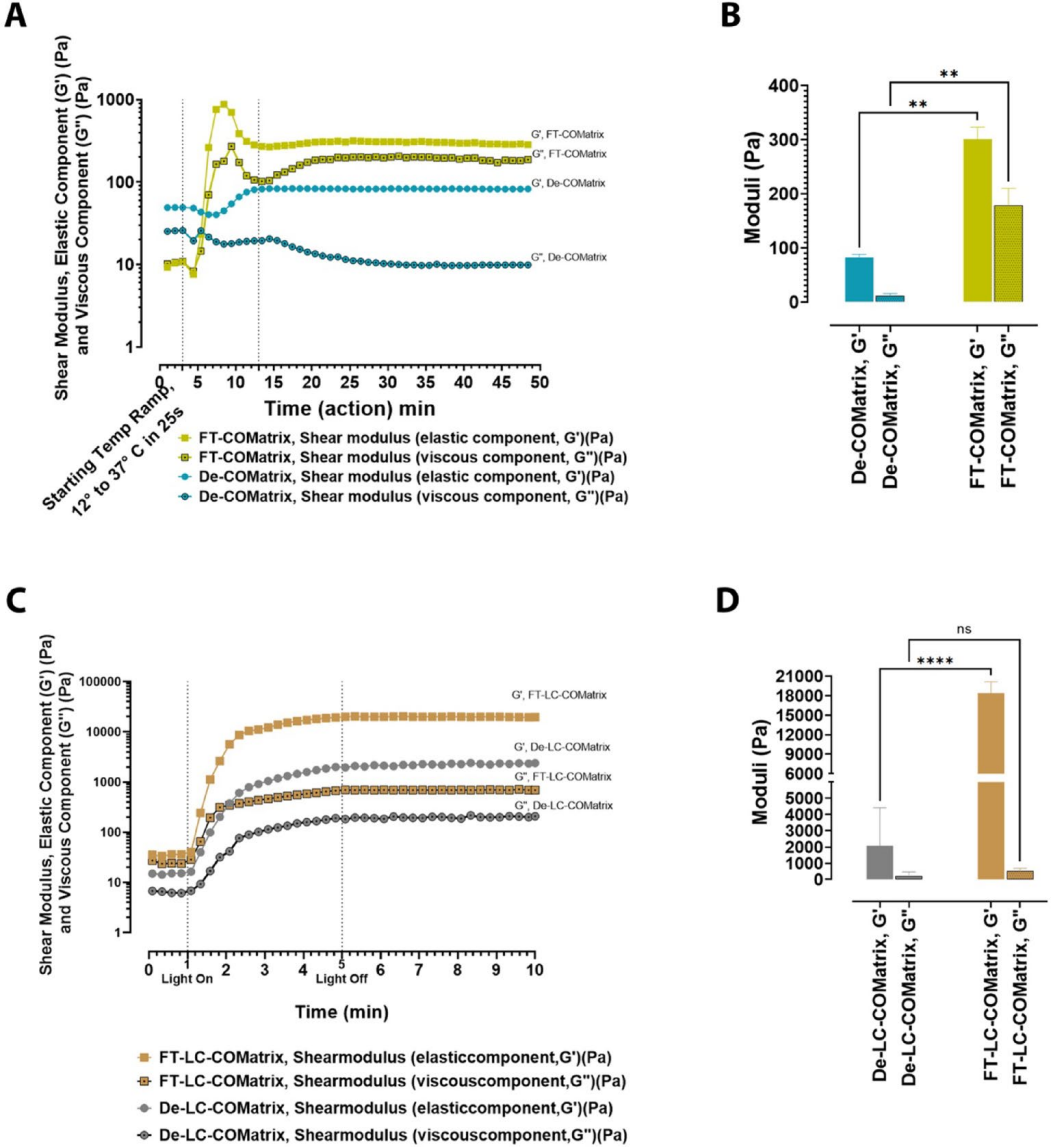
Rheology kinetics and characterization of fabricated COMatrices. (**A**) Rheological kinetics of De-COMatrix and FT-COMatrix thermogelation. (**B**) The elastic (G′) and viscous (G″) shear moduli of thermoresponsive COMatrices. (**C**) Rheological kinetics of De-LC-COMatrix and FT-LC-COMatrix photogelation. (**D**) The elastic (G′) and viscous (G″) shear moduli of light-curable COMatrices. ns, not significant; **, *p* < 0.01; ***, *p* < 0.001; ****, *p* < 0.0001.

We utilized rheology to assess mechanical gelation kinetics and characteristics of the thermoresponsive and light-curable COMatrices. The thermoresponsive hydrogels derived from Detergent-based and freeze–thaw decellularizations were exposed to increasing temperatures from 12 to 37 °C. Higher temperature induced thermogelation, as evident by increase in shear moduli (Fig. 5A), and the thermogelation, which was completed in 10 min. The average final elastic (G′) and viscous (G″ moduli for thermoresponsive De-COMatrix was 82.4 ± 5.5 Pa and 11.1 ± 4.5 Pa, respectively and for thermoresponsive FT-COMatrix was 300.8 ± 22.5 Pa and 178.7 ± 31.3 Pa, respectively. FT-COMatrix hydrogel had significantly higher shear moduli than De-COMatrix hydrogel (N = 3, *p* < 0.01, Fig. 5B).

The photogelation of light-curable COMatrix samples was initiated by turning the light source on and was recorded by increase in the shear moduli (Fig. 5C). The achieved elastic shear moduli (G′) and viscous shear moduli (G″) after 4 min of light curing for FT-LC-COMatrix (25 mg/ml) were 18.3 ± 1.7 kPa and 0.5 ± 0.2 kPa, respectively, and for De-LC-COMatrix (25 mg/ml) were 2.8 ± 2.6 kPa and 0.3 ± 0.2 kPa, respectively. Interestingly, the decellularization method had a significant effect on the biomechanical properties of hydrogels even after functionalizing to become light-curable. The light-curable hydrogels derived from FT decellularization protocol had significantly higher shear moduli compared to hydrogels derived from De decellularization protocol (N = 3, *p* < 0.0001, Fig. 5D).

### Thermoresponsive and light-curable COMatrix hydrogels are cytocompatible

**Figure 6:**
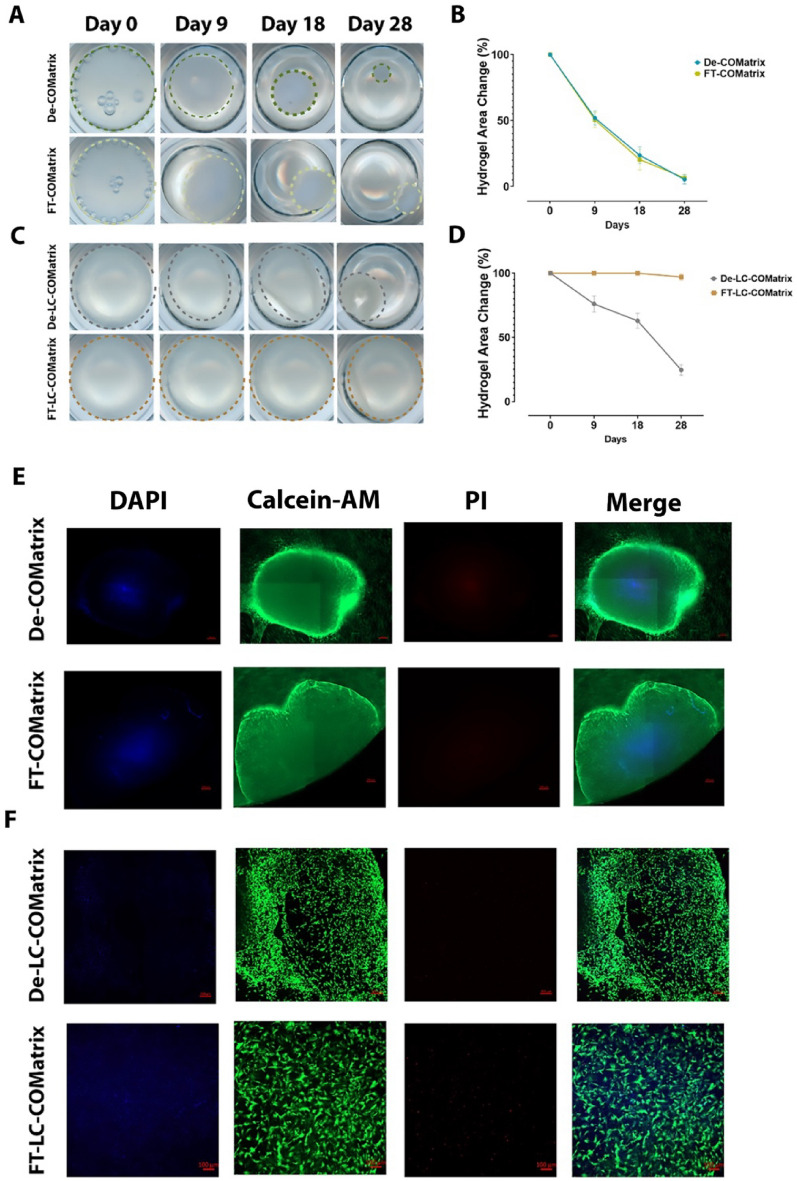
Cytocompatibility of thermoresponsive and light-curable COMatrices after thermogelation and photogelation, respectively. (**A**) Scan of thermoresponsive De- and FT-COMatrices during cell culture with human corneal MSCs from Day 0 to Day 28. (**B**) Changes in the area of thermoresponsive hydrogels (indicating the hydrogel shrinkage) during culture with human corneal MSCs. (**C**) Scan of light-curable De-LC and FT-LC-COMatrices during cell culture with human corneal MSCs from Day 0 to Day 28. (**D**) Changes in the area of light-curbale hydrogels (indicating the hydrogel shrinkage) during culture with human corneal MSCs. Live-dead assay indicating the viability of human corneal MSCs seeded on (**E**) De-COMatrix and FT-COMatrix; and (**F**) De-LC-COMatrix and FT-LC-COMatrix at day 28.

We cultured human corneal mesenchymal stem cells (MSCs) on thermoresponsive COMatrix hydrogels (FT-COMatrix or De-COMatrix) after thermogelation, or on light-curable COMatrix hydrogels (FT-COMatrix or De-COMatrix) after photogelation. The tissue culture plates were scanned on days 0, 9, 18, and 28 to follow the changes in the dimensions of hydrogels. The viability of cells was evaluated at day 28 by using live-dead assay. Over 28 days in culture, the thermoresponsive hydrogels underwent significant shrinkage reflecting the ability of viable MSCs to actively contract the hydrogels (N = 3, Fig. 6A and B), and biomechanical characteristics of thermoresponsive hydrogels. Both thermoresponsive De- and FT-COMatrices shrank significantly and there was no difference between them while comparing their surface area at day 28 of follow-up (*p* = 0.68). The MSCs maintained viability of more than 95% after 28 days in culture on thermoresponsive hydrogels (Fig. 6E).

Light-curable COMatrix was subjected to the same assays revealing notable differences in the biomechanical properties based on the decellularization method. In particular, the detergent-based decellularized light-curable COMatrix hydrogel (De-LC-COMatrix) showed considerable shrinkage on day 28 (24.5 ± 4.1% of primary surface area) whereas freeze–thaw decellularized light-curable COMatrix hydrogel showed significant resistance to cell-mediated contraction during the same period (97.1 ± 2.4% of primary surface area, *p* < 0.0001, Fig. 6C and D). This result is consistent with the rheological data from light-curable hydrogels that revealed higher shear moduli for FT-LC-COMatrix compared to other variants of COMatrix. Live-dead assay performed on day 28 also showed high viability of MSCs on both types of light-curable COMatrices (Fig. 6F).

## Discussion

Hydrogels derived from decellularized porcine corneas show great promise for various ophthalmic therapeutic applications. These hydrogels can be administered in a minimally invasive manner with or without encapsulated cells to enhance corneal wound healing. Modified corneal ECM hydrogels could also be used as a bio-adhesive. This study adds to the previous observations that the decellularization process prior to hydrogel fabrication is crucial and can alter the composition and ultrastructure of the ECM, thus impacting the overall hydrogel characteristics. This study has shown that mechanical strength can be significantly affected by the decellularization method^37^.

Besides maintaining the ECM, the other important objective of the decellularization process is to completely remove cellular antigens that can induce immune responses^11^. There are several methods for removing resident cells, among them a detergent-based decellularization method is shown to be very effective^38^. However, the deleterious effects of detergents upon ECM structure, composition and biochemical properties remains a considerable challenge. Optimum balance between cell removal and the disruption of matrix architecture must be taken into consideration for the clinical usage of *in-situ* hydrogel products^39^.

In this study, we decellularized porcine corneas using a detergent (De) method and a freeze–thaw (FT) method to fabricate thermoresponsive De-COMatrix and FT-COMatrix, respectively. The thermoresponsive hydrogels were further functionalized to become light-curable (LC). Our studies showed that both decellularization processes retained essential ECM components (collagens and sGAGs) while significantly decreasing DNA content. Our data illustrated that both methods could remarkably decrease the α-Gal epitope content. Additionally, we found that treatment with α-galactosidase enzyme significantly reduced the α-Gal epitope. Our analysis of the hydrogels showed that all samples were highly transparent, with no significant difference between the various preparations. The thermoresponsive samples have similar gelation kinetics; however, rheological analysis showed the FT-COMatrix has significantly higher rheological shear moduli than the De-COMatrix. Light-curable COMatrices also showed similar differences as FT-LC-COMatrix is stronger than De-LC-COMatrix. Cytocompatibility evaluations of thermoresponsive and light-curable hydrogels suggested high cell viability in all generated hydrogels. Additionally, LC samples showed significantly less shrinkage compared to thermoresponsive samples when seeded with human corneal MSCs, in particular, FT-LC-COMatrix contracting significantly less than De-LC-COMatrix.

One of the key features of cornea-derived hydrogels is their ability to mimic native tissue, as they retain many of the natural components of the ECM compared to synthetic hydrogels^6^. In previous studies reported by our group and others^31,40,41^, using the freeze–thaw protocol provides superior results over the detergent-based decellularization methods. In this study we used Triton X-100 as a non-ionic detergent as it is a milder reagent compared to ionic detergents like sodium dodecyl sulfate (SDS). In both methods, tissues were cut into small pieces to facilitate the washing and decellularization processes. We showed comparatively similar results between the two methods in terms of cellular removal and preservation of the extracellular matrix. Comparing the two methods, we found similar results in the retention of essential components of decellularized tissue such as collagen and sGAG, keratocan, and removal of DNA from the tissue with minimal disturbance of the basement membrane. Removal of sGAG following decellularization has been previously reported for the cornea^31^. However, our results showed that both collagen and sGAG were retained during both treatment conditions with levels comparable to native porcine cornea. Western blot analysis showed the hydrogels retained the presence of keratocan, a leucine-rich proteoglycan found only in the cornea. Moreover, SEM imaging showed hydrogels retained their porous and fibrillary nature after treatment with both decellularization methods. However, we found a significant difference between the mechanical characterizations of COMatrices derived from detergent-based or freeze–thaw decellularization protocols.

To decrease the chance of rejection of xenogeneic tissues by human hosts, it is critical that all cells and cellular remnants are removed from the decellularized matrix. α-Gal epitope is present in non-primate mammals and is absent in human and apes^42^. The α-Gal epitope remains within the tissue matrix on N-linked oligosaccharides. These epitopes can stimulate the human IgM isotype of anti-Gal which binds to α-Gal epitopes, leading to inflammation, cell lysis via the complement cascade and probable immune-rejection. Therefore, producing an acellular porcine-based ECM graft that preserves extracellular structural and biological integrity of the decellularized organ and simultaneously reduces the antigen-specific response to the α-Gal epitope enables the porcine ECM graft to support and promote regeneration of native tissue. The expression of α-Gal has been shown on pig corneal endothelial cells^28^. Studies have shown that α-galactosidase treatment can effectively remove α-Gal epitope from different organs including porcine heart valve, pericardium and porcine dermal matrix^43–45^, and also bovine pericardial tissue^46^. Our results revealed that De-COMatrix decellularization protocols significantly reduces α-Gal epitope concentration compared to the FT-COMatrix process. However, more profound antigen removal was found when the COMatrix samples were treated with α-galactosidase enzyme.

We analyzed the gelation kinetics of ECM hydrogels by turbidimetric analysis. Previous studies demonstrated considerable differences in turbidimetric data based on decellularization method and showed that freeze–thaw had better turbidimetric gelation^31^. A possible reason for this difference may be due to residual chemicals inherent to the detergent-based methods which is not applicable to freeze–thaw. In our study, after normalization of turbidimetric results, similar sigmoidal profiles (S-shape) for gelation kinetics of both decellularization methods were observed. However, before normalization, the freeze–thaw method showed a more prominent S-shape thermogelation as compared to De-COMatrix. This difference could be the results of more preserved structures in the FT-COMatrix compared to De-COMatrix. On the other hand, there is a slight difference between lag times of De-COMatrix and FT-COMatrix. The previous studies reported lag time differences in hydrogels resulted from different types of decellularization and showed longer lag times in samples decellularized with detergents^31,39^. We believe that the observed difference in t_lag_ (~ 5 min) in this study is not dramatic and since there is no cut-off for significant difference in literature, it is hard to attribute this difference to the decellularization method. Overall, the optimal gelation kinetics should be defined based on the future application of the hydrogel.

Both protocols to produce the COMatrices resulted in similar light transmission. Our analysis of the hydrogels showed that all samples were highly transparent, with no significant difference between the various preparations. However, De-COMatrix is comparatively less transparent compared to FT-COMatrix. This observation is consistent with previous studies showing higher light transmission of hydrogels derived from freeze–thaw decellularization^31^. This difference could be due to detergent remnants in De-COMatrix or more ultra-structure similarity of FT-COMatrix to native cornea as the FT decellularization protocol is less harsh on the corneal tissue.

To evaluate biomechanical properties of hydrogels, rheological analyses were performed. Comparisons of COMatrices obtained from detergent-based and freeze–thaw decellularizations showed that decellularization with a detergent result in significantly weaker hydrogels compared to freeze–thaw decellularization, not only in thermoresponsive hydrogels but also in light-curable COMatrices (functionalized with methacrylate groups). We believe that this difference can be attributed to multiple factors such as more structural disruption, chemical alteration of functional groups like hydroxyl, carboxyl and amine groups, and presence of detergent residues in detergent-based decellularization. The possible alteration in reactive groups by detergents may result in reduction in the degree of functionalization with methacrylate groups in De-LC-COMatrix compared to FT-LC-COMatrix. Moreover, residual detergents could increase the solubility of structural proteins and reduce the surface tension resulting in weaker hydrogels following decellularization with detergents compared to freeze–thaw decellularization^47^. We have observed the structural disruption following decellularization of human cornea with detergents in our previous work^41^.

Cytocompatibility of all the preparations of COMatrices were similar, and none showed cytotoxicity. Differences in biomechanical properties between the four different conditions could be observed with respect to hydrogel shrinkage. Although thermoresponsive FT-COMatrix had significantly higher shear moduli compared to De-COMatrix in rheological measurements, both hydrogels were equally shrunk following seeding MSCs on their surface. This potential paradox could be explained by potential presence of a biomechanical characteristics’ threshold for hydrogel shrinkage. FT-COMatrix and De-COMatrix rheological properties are significantly different; however, both are relatively weak hydrogels which are not reaching the biomechanical characteristics’ threshold for tolerating the contracting effect of MSCs. On the other hand, Hydrogel samples functionalized with methacrylate, to make a photo-initiating cross-linkable hydrogel, showed significantly less shrinkage as compared to their thermoresponsive counterparts, with FT-COMatrix shrinking significantly less than De-COMatrix. Live/dead assays demonstrated that all hydrogel preparations were all highly cytocompatible even after 28 days. This is an important observation for future applications of COMatrix for delivery of mesenchymal stem cells to repair injured corneal stroma.

## Conclusion

In this study, we compared two decellularization methods for fabrication of hydrogels from porcine cornea ECM. First protocol included a series of detergents while the second protocol relied on freeze–thaw cycles. Compositional and gelation kinetic characterizations have not shown significant difference between the COMatrices generated from detergent-based and freeze–thaw decellularization methods. However, significant biomechanical differences were found between the two methods of decellularization even after functionalization of COMatrices to become light-curable. Freeze–thaw decellularization showed superior mechanical features compared to detergent-based decellularization.

## Supplementary Information

Supplementary Figures.

## Data Availability

The datasets generated during the current study are not publicly available due to protection of the intellectual property but are available from the corresponding author on reasonable request.
