# Supplementary material for: The effect of decellularization protocols on characterizations of thermoresponsive and light-curable corneal extracellular matrix hydrogels
Source: Sci Rep. 2023 May 19;13:8145. doi: 10.1038/s41598-023-35202-8 (PMC10199007; doi:10.1038/s41598-023-35202-8)
Supplement: Supplementary file 1 — Supplementary Figures. [file 41598_2023_35202_MOESM1_ESM.docx]

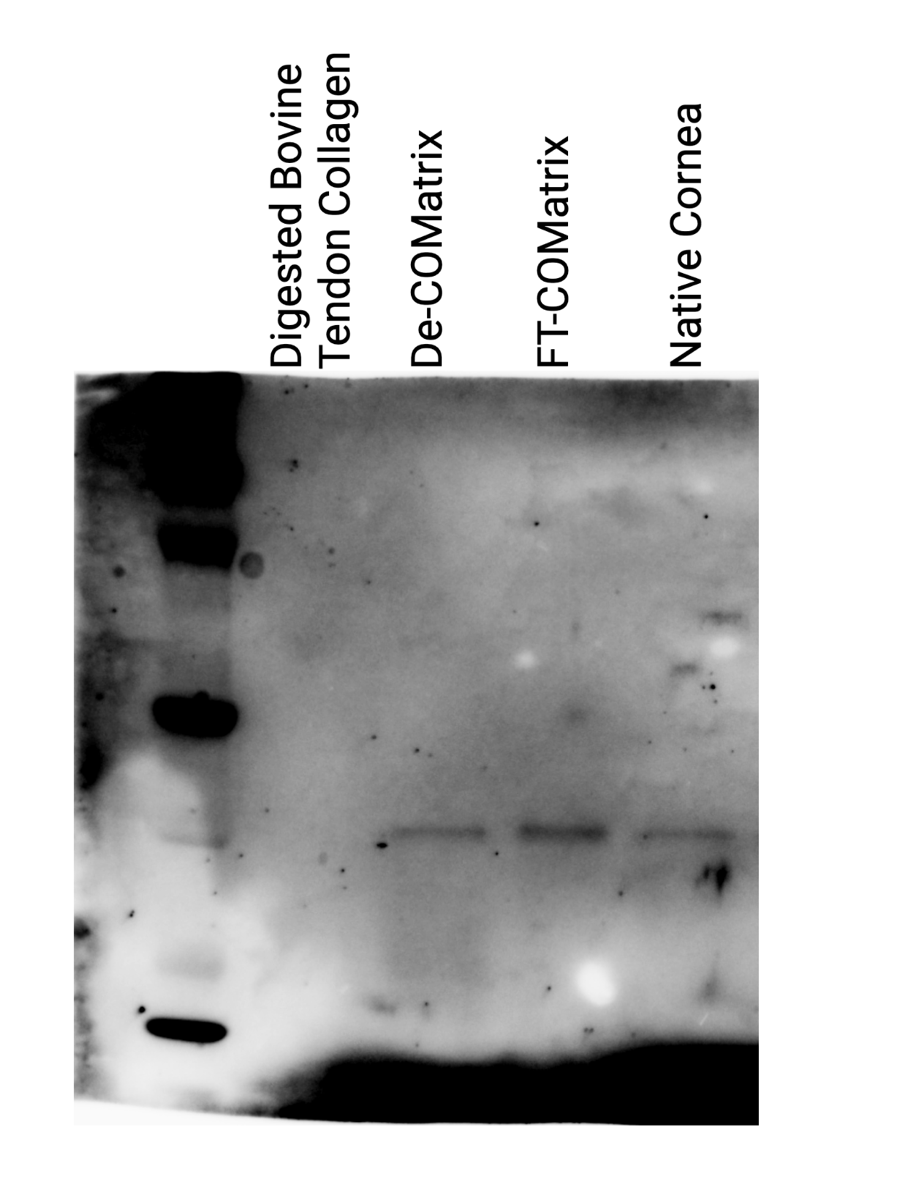


Supplementary Figure 1: Unprocessed image of western blot with Anti-Keratocan Antibody.


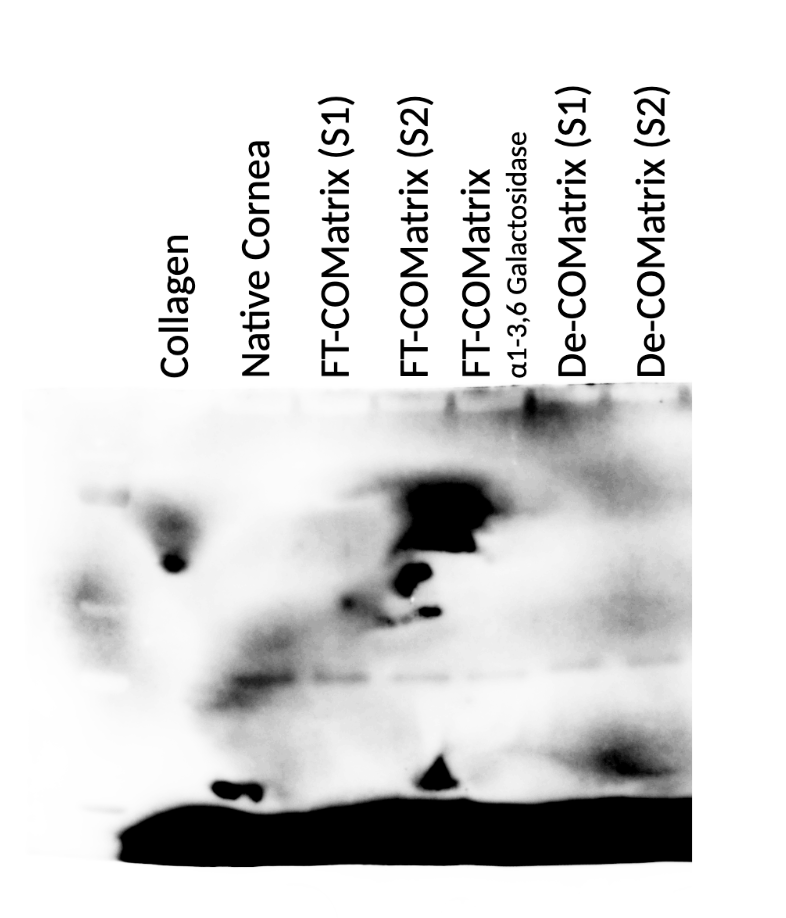


Supplementary Figure 2: Unprocessed image of western blot with Anti-α-Gal epitope Antibody.
